# Philadelphia Correspondence

**Published:** 1868-12-01

**Authors:** 


### PHILADELPHIA CORRESPONDENCE.

Philadelphia, November 12, 1868.

Editor Chicago Medical Journal: As I mentioned in my last communication to your columns, as probably to take place, the reception to Professors Gross and Pancoast came off at the Foyer of the Academy of Music on Saturday evening, October 25, and it was a magnificent affair. Many of the distinguished medical gentlemen were present from other cities, and it was a brilliant entertainment, well conducted, and eminently well merited.

I shall close my short series of letters on the subject of uterine diseases with this letter, noticing in the first place the *hypertrophied uterus.* It is impossible to find an ulcerated, or indeed even an inflamed uterus, without hypertrophy more or less marked. But there are cases of enlargement without any present ulceration or inflammation even. I have noted sixty-five cases of hypertrophied uteri with no other form of disease existing, and as a natural sequence, more or less prolapse in all. The womb increasing in all its dimensions, necessarily increases its weight. This augmentation of weight forces the uterus down into the vagina, notwithstanding the vaginal walls may be quite tense, and the patient presents herself for treatment for "falling of the womb." How shall we treat this? By pessaries? By caustics? Better than pessaries, but better than all, by sponge tents. They *may* fail, but in the sixty-five cases before mentioned, in forty-one the sponge tents were all the treatment. Tent after tent was introduced, and in thirty-seven the cure was complete. So far as our observation extends, the larger proportion of prolapsed uteri are due to hypertrophy, and when this is cured the prolapse is cured.

In hypertrophied and in inflamed and abraded uteri, the knife has been recommended by Louis especially, and he advised the cervix in many cases — especially in occluded os uteri — to be incised. In our clinic, and in my own private practice, the mucous membrane lining the cavity of cervix, as well as the mucous covering of the os, has been freely scarified by bistoury, with the most astonishingly rapid improvement. Incisions — several in number — are made in all directions, remembering the great thickness of cervical mucous membrane, and blood allowed to freely exude. Oftentimes an hypertrophy will melt away, and congestion disappear before the patient rises from the bed. I would strongly urge it as a plan worthy of very general use. As I have before remarked, the pathology, as well as treatment, of uterine diseases, are rapidly progressing. It is a specialty demanding the attention and study of the general practitioner of the country, for to him will fall a large amount of uterine diseases, which, if properly managed, will be the best tests and proofs of the value of any suggestion I may have dropped in these letters.

If I have aided one mind upon this subject, through the large circulation of as valuable journal as yours, I shall be well satisfied.

I have an interesting case of a large uterine polypus to present you with in my next. Yours, etc.,

